# Supplementary material for: Plasmodium falciparum GBP2 Is a Telomere-Associated Protein That Binds to G-Quadruplex DNA and RNA
Source: Front Cell Infect Microbiol. 2022 Feb 22;12:782537. doi: 10.3389/fcimb.2022.782537 (PMC8902816; doi:10.3389/fcimb.2022.782537)
Supplement: Supplementary file 1 [file DataSheet_1.pdf]

SUPPLEMENTARY FIGURES AND TABLES

S1 Figure. ChIP on *Pf*GBP2

(A) Dotblots of chromatin immunoprecipitated with antibodies to *Pf*GBP2-3HA or to histone H4. Fold enrichment over input is shown. Telomere sequence is enriched by ~2x with antibody to *Pf*GBP2-HA as well as antibody to histone H4, whereas a control non-telomeric gene is detected only in histone ChIP and is not enriched in HA ChIP.
(B) Examples of ChIP data from the *Pf*GBP2-Ty line which co-expresses *Pf*HP1-HA. Genome-wide ChIP-seq conducted in duplicate on this line, and also on the *Pf*GBP2-3HA line, failed to give signals significantly above background (data not shown). To confirm the validity of the ChIP procedure using the validated chromatin protein *Pf*HP1 as a control, ChIP was conducted with a selection of individual primer pairs directed to telomere repeats, subtelomeric genes, G-quadruplex-encoding genes, or chromosome-internal genes. Representative data from one of two duplicate experiments are shown. *Pf*HP1-HA, as expected, was enriched by >50-fold at sub-telomeric loci compared to chromosome-internal loci when it was immunoprecipitated with either anti-HA-tag or anti-HP1 antibodies. By contrast, *Pf*GBP2-Ty, present in the same parasites, was not strongly enriched at any locus: the mean enrichment at sub-telomeric and G4-encoding genes compared to non-telomeric genes was only 2.4-fold.

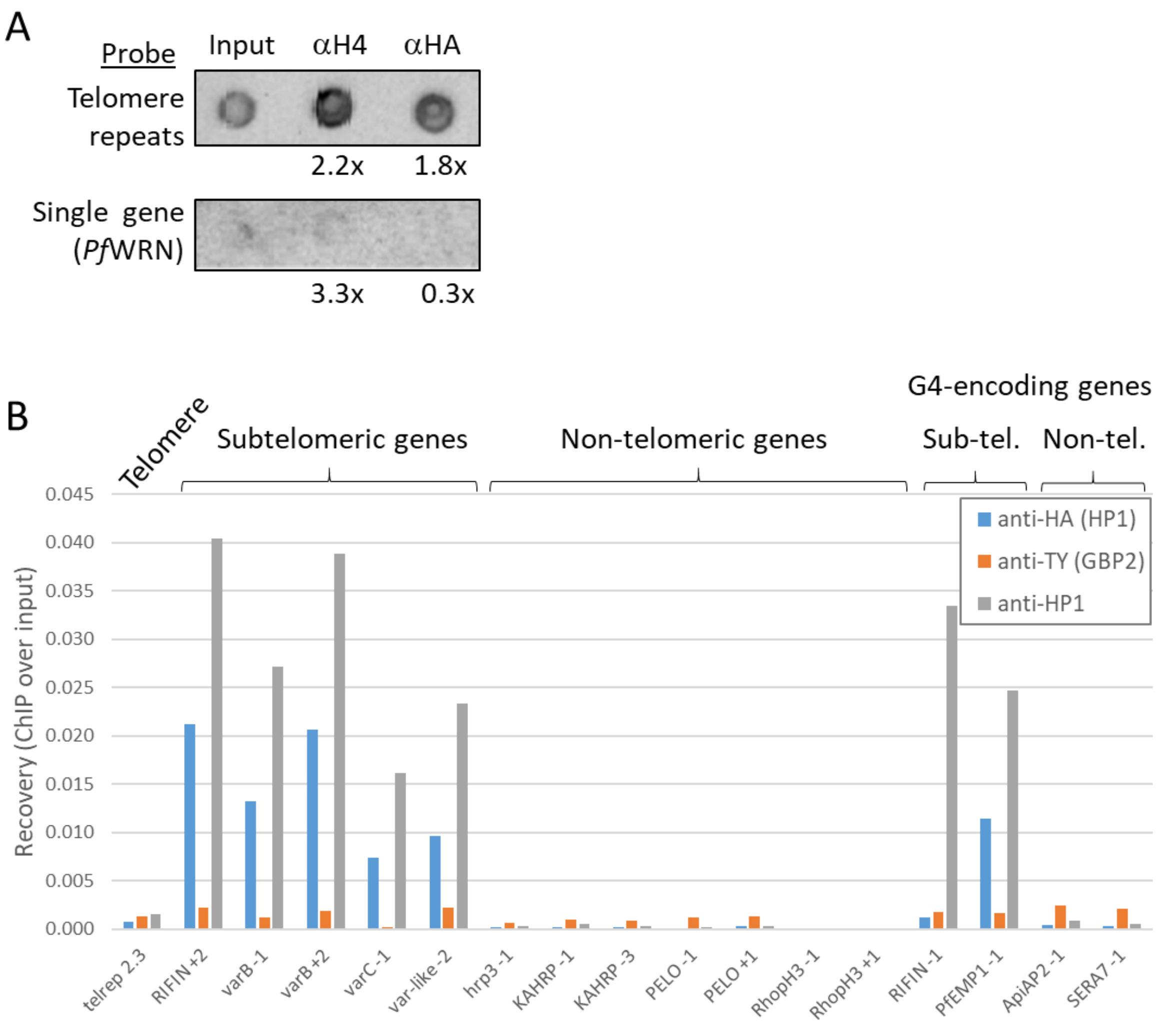

S1 Table*.* Protein sequence matching data from FASP and GASP analysis of PICh extracts.

Control (Ctl) and Target data from FASP (experiment 1) are compared to a second dataset obtained via GASP (experiment 2) using Scaffold, with a 95% threshold for proteins and peptides, and a minimum of two peptides/hit. 'Exclusive Unique Peptide Count' is shown for each sample. Searches were performed using Mascot against a hybrid database containing sequences derived from *Plasmodium falciparum* (download date 20th July 2015), combined with common contaminant proteins (cRAP, download date 30th January 2015 and contaminants, download date 13th July 2012, both downloaded from Matrix Science). No *Plasmodium* peptides were observed in the control GASP experiment, although contaminating proteins were detected. This is possibly due to a technical error, but some *Plasmodium* peptides were detected in the preceding FASP control experiment.

S2 Table*.* Table of oligonucleotide sequences

In PICh probes, 'C18' refers to the flexible linker region. Locked Nucleic Acid (LNA) bases are in capitals. 'y' is a 67% T, 33% C custom base mix, designed to mimic the variable position in the *Plasmodium* telomere repeat (GGGTT(T/C)A) where the ratio is approx. 67% T: 33%C.

S3 Table*.* Proteins immunoprecipitated with *Pf*GBP2

Tab 1 shows all protein hits from duplicate anti-HA immunoprecipitations of *Pf*GBP2-3HA, after any proteins also found in a control IP experiment from wildtype parasites have been screened out. Tab 2 shows the subset of proteins that were reproducibly found in both *Pf*GBP2-3HA immunoprecipitations. Tab 3 shows proteins found uniquely in the control IP. GO terms for each hit are listed in the classes 'cellular component', 'biological function' and 'molecular process'. The mass spectrometry data have been deposited to the ProteomeXchange Consortium via the PRIDE partner repository with the dataset identifier PXD028903.

S4 Table*.* GO terms enriched amongst proteins immunoprecipitated with *Pf*GBP2

Enriched GO terms in the classes 'cellular component', 'biological function' and 'molecular process' are shown for the reproducible *Pf*GBP2 interactors, and also for total set of *Pf*GBP2 interactors found in at least one IP experiment. GO terms with statistically significant enrichment after correction for multiple comparisons are highlighted in yellow. Terms associated with RNA are in red text, and with DNA in blue text.
